# The Role of Three-Dimensional Printing in Thoracic and Cardiovascular Surgery: Setting a Milestone in Peru

**DOI:** 10.21470/1678-9741-2023-0089

**Published:** 2023-09-19

**Authors:** Franco Alejandro Albán Sánchez, Wildor Samir Cubas Llalle

**Affiliations:** 1 Department of Thoracic and Cardiovascular Surgery, Edgardo Rebagliati Martins National Hospital, Lima, Peru; 2 Department of Vascular and Endovascular Surgery Service, Edgardo Rebagliati Martins National Hospital, Lima, Peru

Dear Editor,

Three-dimensional (3D) printing has attracted the medical world’s attention throughout the last few years. It has presented a fast development in clinical and surgical medical practice since its introduction in the 1980s. Since then, this technological tool has shown great utility in multiple fields, especially in surgery, due to the possibility of transforming digital information into physical models, making it possible to achieve a more objective surgical analysis and planning, especially in complex anatomies^[1]^.

Lately, many studies have demonstrated the usefulness of 3D printing, not only for preoperative planning, simulation, and intraoperative guidance but also for teaching and research, as well as having the potential to provide, in the future, prosthetic biological impressions or even transplantation material^[2]^. Current evidence has reported that its use in thoracic and cardiovascular surgery reduces operative time by up to 19.85% and decreases the intraoperative error rate by approximately 2.9%, consequently increasing the efficiency of procedures by 3.6% and optimizing the surgeon’s learning curve. Thus, 3D printing has been proven to be a helpful complement to the usual image-based planning, achieving a safe surgery with lower morbimortality and better results^[3]^.

In Peru, 3D impressions are being used more and more frequently in orthopedic and general surgery specialties; however, before the introduction of this technology, surgical planning was based on the interpretation and reconstruction of radiological images only, which allowed us to capture human anatomy subjectively. This interpretation was sometimes over- or undersized, which meant a potential risk of measurement errors and, therefore, less accurate and detailed planning.

The surgical program of Thoracic and Cardiovascular Surgery in our institution has been developing recently, and, more frequently, 3D printed models are being used for planning, orientation, teaching, research, and training of surgical teams. In the thoracic surgery field, we have made reconstructions of the central airway and bronchial tree of patients when information regarding the exact anatomy, orientation, and dimensions is required to perform a better approach with fewer complications. Concerning heart surgery, real-scale models of the great vessels and surgical cardiac anatomy have been used to get cannulation and connection strategies to the heart-lung machine. These also have been used to obtain more precise dimensions in cases of aneurysmal dilatations of the aorta and pulmonary vessels. However, the real benefit takes place in the improvement of endovascular and open vascular surgery, allowing the implementation of life-size printed models in preoperative planning, making procedures such as endovascular repair of abdominal aortic aneurysms, thoracic endovascular aortic repair, and repair with branched devices successful (Figure 1).

**Fig. 1:**
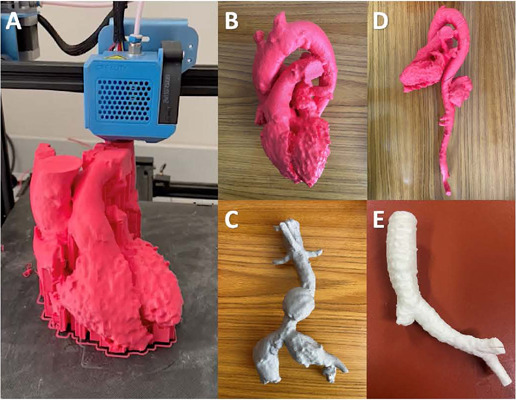
Three-dimensional printing models. (A) Printing process of the heart and great vessels at a 1:1 scale. (B) Heart panoramic view with its chambers and great vessels (C) Abdominal aorta with an infrarenal fusiform aneurysm and bilateral aneurysm of common iliac vessels. (D) Heart, great vessels, and whole aorta with a saccular aneurysm in the thoracic segment. (E) Trachea segment with a bronchopleural fistula on a short right stump in a postpneumonectomy patient.

This proposal seeks to improve surgical services in Peru, enhance the skills and clinical-surgical analysis of medical professionals, and, above all, give the initiative for all Peruvian hospitals to imitate and adopt the tools used in developed countries and which have been generating good results, with a broad future for the growth of this technology, always looking for the benefit of patients.

## References

[1] Kwok JKS, Lau RWH, Zhao ZR, Yu PSY, Ho JYK, Chow SCY (2018). Multi-dimensional printing in thoracic surgery: current and
future applications. J Thorac Dis.

[2] Witowski J, Sitkowski M, Zuzak T, Coles-Black J, Chuen J, Major P (2018). From ideas to long-term studies: 3D printing clinical trials
review. Int J Comput Assist Radiol Surg.

[3] Montanhesi PK, Coelho G, Curcio SAF, Poffo R (2022). Three-dimensional printing in minimally invasive cardiac surgery:
optimizing surgical planning and education with life-like
models. Braz J Cardiovasc Surg.

